# Identification of constrained sequence elements across 239 primate genomes

**DOI:** 10.1038/s41586-023-06798-8

**Published:** 2023-11-29

**Authors:** Lukas F. K. Kuderna, Jacob C. Ulirsch, Sabrina Rashid, Mohamed Ameen, Laksshman Sundaram, Glenn Hickey, Anthony J. Cox, Hong Gao, Arvind Kumar, Francois Aguet, Matthew J. Christmas, Hiram Clawson, Maximilian Haeussler, Mareike C. Janiak, Martin Kuhlwilm, Joseph D. Orkin, Thomas Bataillon, Shivakumara Manu, Alejandro Valenzuela, Juraj Bergman, Marjolaine Rouselle, Felipe Ennes Silva, Lidia Agueda, Julie Blanc, Marta Gut, Dorien de Vries, Ian Goodhead, R. Alan Harris, Muthuswamy Raveendran, Axel Jensen, Idriss S. Chuma, Julie E. Horvath, Christina Hvilsom, David Juan, Peter Frandsen, Joshua G. Schraiber, Fabiano R. de Melo, Fabrício Bertuol, Hazel Byrne, Iracilda Sampaio, Izeni Farias, João Valsecchi, Malu Messias, Maria N. F. da Silva, Mihir Trivedi, Rogerio Rossi, Tomas Hrbek, Nicole Andriaholinirina, Clément J. Rabarivola, Alphonse Zaramody, Clifford J. Jolly, Jane Phillips-Conroy, Gregory Wilkerson, Christian Abee, Joe H. Simmons, Eduardo Fernandez-Duque, Sree Kanthaswamy, Fekadu Shiferaw, Dongdong Wu, Long Zhou, Yong Shao, Guojie Zhang, Julius D. Keyyu, Sascha Knauf, Minh D. Le, Esther Lizano, Stefan Merker, Arcadi Navarro, Tilo Nadler, Chiea Chuen Khor, Jessica Lee, Patrick Tan, Weng Khong Lim, Andrew C. Kitchener, Dietmar Zinner, Ivo Gut, Amanda D. Melin, Katerina Guschanski, Mikkel Heide Schierup, Robin M. D. Beck, Ioannis Karakikes, Kevin C. Wang, Govindhaswamy Umapathy, Christian Roos, Jean P. Boubli, Adam Siepel, Anshul Kundaje, Benedict Paten, Kerstin Lindblad-Toh, Jeffrey Rogers, Tomas Marques Bonet, Kyle Kai-How Farh

**Affiliations:** 1 Illumina Artificial Intelligence Laboratory, Illumina, San Diego, CA USA; 2 UC Santa Cruz Genomics Institute, University of California, Santa Cruz, CA USA; 3 Science for Life Laboratory, Department of Medical Biochemistry and Microbiology, Uppsala University, Uppsala, Sweden; 4 School of Science, Engineering and Environment, University of Salford, Salford, UK; 5 Department of Evolutionary Anthropology, University of Vienna, Vienna, Austria; 6 Human Evolution and Archaeological Sciences (HEAS), University of Vienna, Vienna, Austria; 7 Département d’Anthropologie, Université de Montréal, Montréal, Quebec Canada; 8 Bioinformatics Research Centre, Aarhus University, Aarhus, Denmark; 9 Academy of Scientific and Innovative Research (AcSIR), Ghaziabad, India; 10 Laboratory for the Conservation of Endangered Species, CSIR-Centre for Cellular and Molecular Biology, Hyderabad, India; 11 IBE, Institute of Evolutionary Biology (UPF-CSIC), Department of Medicine and Life Sciences, Universitat Pompeu Fabra, Barcelona, Spain; 12 Section for Ecoinformatics and Biodiversity, Department of Biology, Aarhus University, Aarhus, Denmark; 13 Research Group on Primate Biology and Conservation, Mamirauá Institute for Sustainable Development, Tefé, Brazil; 14 Evolutionary Biology and Ecology (EBE), Département de Biologie des Organismes, Université libre de Bruxelles (ULB), Brussels, Belgium; 15 Centro Nacional de Analisis Genomico (CNAG), Barcelona, Spain; 16 Human Genome Sequencing Center and Department of Molecular and Human Genetics, Baylor College of Medicine, Houston, TX USA; 17 Department of Ecology and Genetics, Animal Ecology, Uppsala University, Uppsala, Sweden; 18 Tanzania National Parks, Arusha, Tanzania; 19 North Carolina Museum of Natural Sciences, Raleigh, NC USA; 20 Department of Biological and Biomedical Sciences, North Carolina Central University, Durham, NC USA; 21 Department of Biological Sciences, North Carolina State University, Raleigh, NC USA; 22 Department of Evolutionary Anthropology, Duke University, Durham, NC USA; 23 Renaissance Computing Institute, University of North Carolina at Chapel Hill, Chapel Hill, NC USA; 24 Copenhagen Zoo, Frederiksberg, Denmark; 25 Universidade Federal de Viçosa, Viçosa, Brazil; 26 Universidade Federal do Amazonas, Departamento de Genética, Laboratório de Evolução e Genética Animal (LEGAL), Manaus, Brazil; 27 Department of Anthropology, University of Utah, Salt Lake City, UT USA; 28 Universidade Federal do Para, Bragança, Brazil; 29 Research Group on Terrestrial Vertebrate Ecology, Mamirauá Institute for Sustainable Development, Tefé, Brazil; 30 Rede de Pesquisa em Diversidade, Conservação e Uso da Fauna da Amazônia – RedeFauna, Manaus, Brazil; 31 Comunidad de Manejo de Fauna Silvestre en la Amazonía y en Latinoamérica—ComFauna, Iquitos, Peru; 32 Universidade Federal de Rondônia, Porto Velho, Brazil; 33 Instituto Nacional de Pesquisas da Amazônia, Manaus, Brazil; 34 Instituto de Biociências, Universidade Federal do Mato Grosso, Cuiabá, Brazil; 35 Department of Biology, Trinity University, San Antonio, TX USA; 36 Life Sciences and Environment, Technology and Environment of Mahajanga, University of Mahajanga, Mahajanga, Madagascar; 37 Department of Anthropology, New York University, New York, NY USA; 38 Department of Neuroscience, Washington University School of Medicine in St Louis, St Louis, MO USA; 39 Keeling Center for Comparative Medicine and Research, MD Anderson Cancer Center, Bastrop, TX USA; 40 Department of Anthropology, Yale University, New Haven, CT USA; 41 School of Interdisciplinary Forensics, Arizona State University, Phoenix, AZ USA; 42 California National Primate Research Center, University of California, Davis, CA USA; 43 Guinea Worm Eradication Program, The Carter Center Ethiopia, Addis Ababa, Ethiopia; 44 State Key Laboratory of Genetic Resources and Evolution, Kunming Institute of Zoology, Chinese Academy of Sciences, Kunming, China; 45 Center for Evolutionary and Organismal Biology, Zhejiang University School of Medicine, Hangzhou, China; 46 Villum Centre for Biodiversity Genomics, Section for Ecology and Evolution, Department of Biology, University of Copenhagen, Copenhagen, Denmark; 47 Liangzhu Laboratory, Zhejiang University Medical Center, Hangzhou, China; 48 Women’s Hospital, School of Medicine, Zhejiang University, Hangzhou, China; 49 Tanzania Wildlife Research Institute (TAWIRI), Arusha, Tanzania; 50 Institute of International Animal Health/One Health, Friedrich-Loeffler-Institut, Federal Research Institute for Animal Health, Greifswald–Insel Riems, Germany; 51 Professorship for International Animal Health/One Health, Faculty of Veterinary Medicine, Justus Liebig University, Giessen, Germany; 52 Department of Environmental Ecology, Faculty of Environmental Sciences, University of Science and Central Institute for Natural Resources and Environmental Studies, Vietnam National University, Hanoi, Vietnam; 53 Institut Català de Paleontologia Miquel Crusafont, Universitat Autònoma de Barcelona, Barcelona, Spain; 54 Department of Zoology, State Museum of Natural History Stuttgart, Stuttgart, Germany; 55 Institució Catalana de Recerca i Estudis Avançats (ICREA), Barcelona, Spain; 56 Centre for Genomic Regulation (CRG), The Barcelona Institute of Science and Technology, Barcelona, Spain; 57 Barcelonaβeta Brain Research Center, Pasqual Maragall Foundation, Barcelona, Spain; 58 Universitat Pompeu Fabra, Barcelona, Spain; 59 Cuc Phuong Commune, Nho Quan District, Vietnam; 60 Genome Institute of Singapore, Agency for Science, Technology and Research, Singapore, Singapore; 61 Mandai Nature, Singapore, Singapore; 62 SingHealth Duke–NUS Institute of Precision Medicine (PRISM), Singapore, Singapore; 63 Cancer and Stem Cell Biology Program, Duke-NUS Medical School, Singapore, Singapore; 64 SingHealth Duke–NUS Genomic Medicine Centre, Singapore, Singapore; 65 Department of Natural Sciences, National Museums Scotland, Edinburgh, UK; 66 School of Geosciences, Edinburgh, UK; 67 Cognitive Ethology Laboratory, Germany Primate Center, Leibniz Institute for Primate Research, Göttingen, Germany; 68 Department of Primate Cognition, Georg-August-Universität Göttingen, Göttingen, Germany; 69 Leibniz ScienceCampus Primate Cognition, Göttingen, Germany; 70 Department of Anthropology and Archaeology, University of Calgary, Calgary, Alberta Canada; 71 Department of Medical Genetics, University of Calgary, Calgary, Alberta Canada; 72 Alberta Children’s Hospital Research Institute, University of Calgary, Calgary, Alberta Canada; 73 Institute of Ecology and Evolution, School of Biological Sciences, University of Edinburgh, Edinburgh, UK; 74 Cardiovascular Institute, Stanford University, Stanford, CA USA; 75 Department of Cardiothoracic Surgery, Stanford University, Stanford, CA USA; 76 Department of Cancer Biology, Stanford University, Stanford, CA USA; 77 Department of Dermatology, Stanford University School of Medicine, Stanford, CA USA; 78 Veterans Affairs Palo Alto Healthcare System, Palo Alto, CA USA; 79 Gene Bank of Primates and Primate Genetics Laboratory, German Primate Center, Leibniz Institute for Primate Research, Göttingen, Germany; 80 Simons Center for Quantitative Biology, Cold Spring Harbor Laboratory, Cold Spring Harbor, NY USA; 81 Department of Computer Science, Stanford University, Stanford, CA USA; 82 Department of Genetics, Stanford University, Stanford, CA USA; 83 Broad Institute of MIT and Harvard, Cambridge, MA USA

**Keywords:** Comparative genomics, Evolutionary genetics, Genetic variation, Transcriptional regulatory elements, Genome evolution

## Abstract

Noncoding DNA is central to our understanding of human gene regulation and complex diseases^1,2^, and measuring the evolutionary sequence constraint can establish the functional relevance of putative regulatory elements in the human genome^3–9^. Identifying the genomic elements that have become constrained specifically in primates has been hampered by the faster evolution of noncoding DNA compared to protein-coding DNA^10^, the relatively short timescales separating primate species^11^, and the previously limited availability of whole-genome sequences^12^. Here we construct a whole-genome alignment of 239 species, representing nearly half of all extant species in the primate order. Using this resource, we identified human regulatory elements that are under selective constraint across primates and other mammals at a 5% false discovery rate. We detected 111,318 DNase I hypersensitivity sites and 267,410 transcription factor binding sites that are constrained specifically in primates but not across other placental mammals and validate their *cis*-regulatory effects on gene expression. These regulatory elements are enriched for human genetic variants that affect gene expression and complex traits and diseases. Our results highlight the important role of recent evolution in regulatory sequence elements differentiating primates, including humans, from other placental mammals.

## Abstract (web summary)

Whole-genome alignment of 239 primate species reveals noncoding regulatory elements that are under selective constraint in primates but not in other placental mammals, that are enriched for variants that affect human gene expression and complex traits in diseases.

## Main

Functional genomic elements that have acquired selective constraint specific to the primate order are prime candidates for understanding the evolutionary changes that have contributed to the uniqueness of our own species^13–16^. Whereas comparisons between the human genome and those of other mammal and vertebrate species have revealed an extensive catalogue of constrained genes and regulatory elements^4–6,17,18^, identifying constrained sequence elements that are specific to primates has been particularly challenging owing to the short evolutionary distances separating these species^5,18^. Compared with the mammalian lineage, which includes more than 6,000 species separated by more than 100 million years of evolution^19^, the primate order only consists of approximately 500 species that are separated by a fraction of this time^11^—around 65 million years. Thus, despite 43 primate species having been aligned in the recent Zoonomia study^20^ of 240 placental mammals, the total phylogenetic branch length within these primates is only around 10% that of the placental mammal alignment^21^. At such short timescales, it is unclear whether the absence of genetic changes between species is owing to functional constraints, or simply because insufficient time has passed for random mutations to arise. Consequently, the selective constraints specific to the phylogenetic branch from which the human species ultimately emerged remain largely unidentified.

We recently reported a catalogue of genetic diversity in primates based on hundreds of species and individuals, which enabled us to gain insight into evolutionary and population dynamics in the primate order^11,22^. Leveraging the vast new catalogue of benign missense mutations in these species, we further developed and applied models to identify pathogenic variants in protein-coding sequences, which account for only 1% of the human genome^23,24^. Here, we expand on these prior works by constructing a genome-wide multiple sequence alignment (MSA) of 239 primate species to better characterize constraint at noncoding regulatory sequences in the human genome. Using comparisons with other mammals, we identify an important class of noncoding regulatory elements with constraint specific to primates and delineate a role for these elements in human health by integrating functional genomics and population genetics datasets.

## A 239-way primate whole-genome alignment

**Fig. 1:**
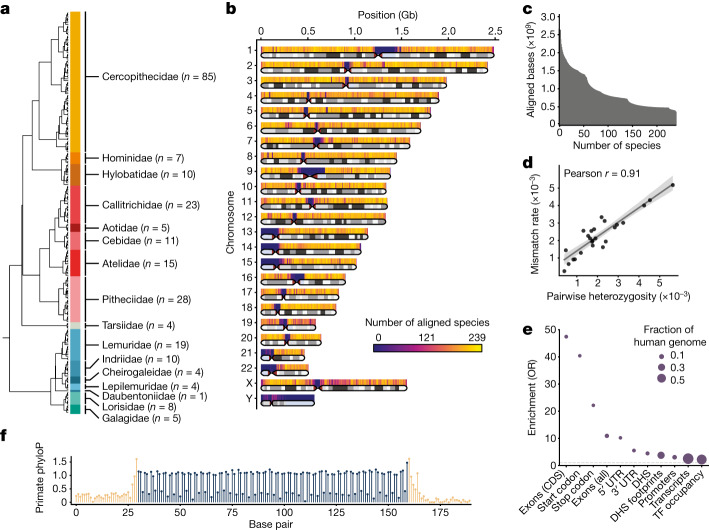
MSA of 239 primate species. **a**, Cladogram of primate species included in the MSA. The number of sampled species per family is given in parenthesis. **b**, Ideogram of the human genome depicting the average number of species covered by the MSA at 500-kb resolution. Telomeric, centromeric and heterochromatic regions (light blue) are indicated. **c**, Cumulative primate species coverage of the human genome in the 239-way primate MSA. **d**, Per-base mismatch rate between newly generated short-read contigs and species with previously published high-quality reference assemblies. A linear regression fit with a corresponding 95% confidence interval ribbon is shown. **e**, Enrichment of primate phastCons elements for coding and noncoding genomic elements. The size of the circle represents the fraction of the human genome. The dashed grey line indicates an odds ratio (OR) of 1. CDS, coding sequence; TF, transcription factor; UTR, untranslated region. **(f)** Codon periodicity in the mean primate phyloP scores across 482 protein-coding exons exactly 130 nucleotides in length. Coding sequences are shown in dark blue and flanking intronic sequences in beige.

To identify genomic elements with primate-specific constraint, we constructed a multiple sequence alignment that densely samples the primate lineage. We identified 187 primate species without an available reference assembly that had recently reported Illumina whole-genome sequencing data^11,23^, and assembled their genomes using Megahit^25^ based on an average coverage of 35× per individual. We combined the resulting contigs together with 52 previously published high-quality primate reference assemblies to create a reference-free whole-genome MSA of 239 primate species with Cactus^21^ (Supplementary Data 1). This alignment represents all major primate lineages, including 86% of genera and all 16 families (Fig. 1a,b). As our goal was to quantify sequence constraint across the human genome, we confirmed that each base was covered by an average of 174 other primate species, and 85% percent of the euchromatic regions of the human genome were covered by at least 100 other primate species (Fig. 1c). To ensure that the per-base error rate in our de novo assemblies was sufficiently low for subsequent constraint analysis, we compared a set of 25 species within our data for which both newly generated short-read contigs and previously published reference genomes were available. We found that the rates of mismatches between these assembly pairs ranged between 0.02 and 0.5% and were largely explained by differences in the species’ heterozygosity (Fig. 1d and Supplementary Table 1). After accounting for intraspecific variation, the average remaining mismatch rate attributable to assembly and sequencing errors was reduced to 0.04% (Methods). Finally, we generated a 441-species mammalian MSA by combining our primate MSA with the remaining mammalian orders sampled in Zoonomia^20^. This constitutes the deepest species sampling for mammals in a whole-genome MSA to date, including 204 primate species unique to this study, and enables detection of sequence constraint both broadly across mammals and in the more recent evolution of our own lineage.

## Primate-constrained protein-coding sequences

**Extended Data Fig. 1:**
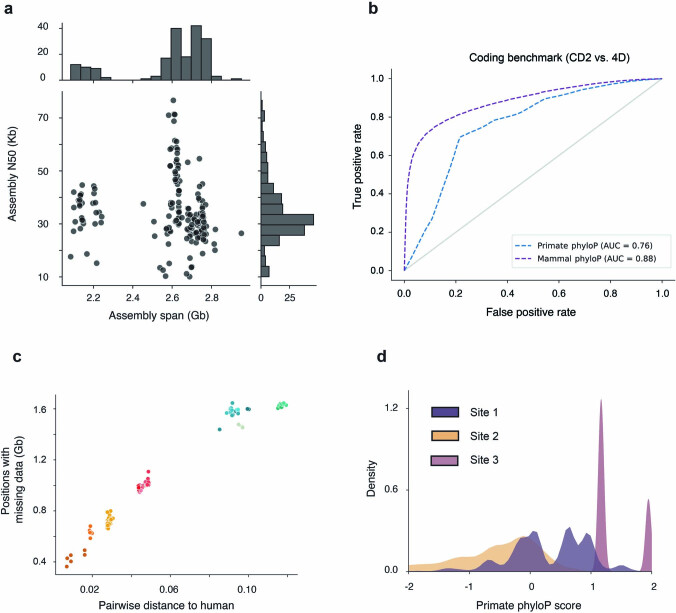
Genome assemblies and constraint metrics. **(a)** Distribution of genome assembly span and contiguity for newly assembled primate species in this project. The cluster with assembly spans <2.3 Gb corresponds to Strepsirrhines, which have smaller genomes sizes then remaining primate species. **(b)** ROC-curves for coding benchmark across mammal and primate phyloP, comparing codon positions 2 (CD2) as putatively constrained positive cases, and human four-fold degenerate sites (4D) as negative cases. Both primate and mammal phyloP distinguish well between non-synonymous CD2 and 4-fold degenerate sites, while mammal phyloP achieves expectedly higher performance due to the larger total branch-length covered by the MSA. **(c)** Scatterplot showing the proportion of bases in the human genome with missing data in the filtered MSA, after excluding ambiguous alignments and duplications for a given species, versus the pairwise phylogenetic distance to human. The proportion of resolved bases has a strong phylogenetic clustering, points are colored by the corresponding primate family following the color scheme presented in Fig. 1a. **(d)** Effect of alignment composition on phyloP scores for 3 different scenarios: Site 1 contains positions with perfectly matching alignments in 151-171 species and missing alignments in the remaining ones, Site 2 contains positions with perfectly matching alignments in 151-171 species but mismatches in over 50 species, Site 3 contains perfect alignments across all species. Distributions for Site1 and Site 2 are significantly different (P = 1.4 × 10^−66^, two-sided Rank Sum Test).

**Extended Data Fig. 2:**
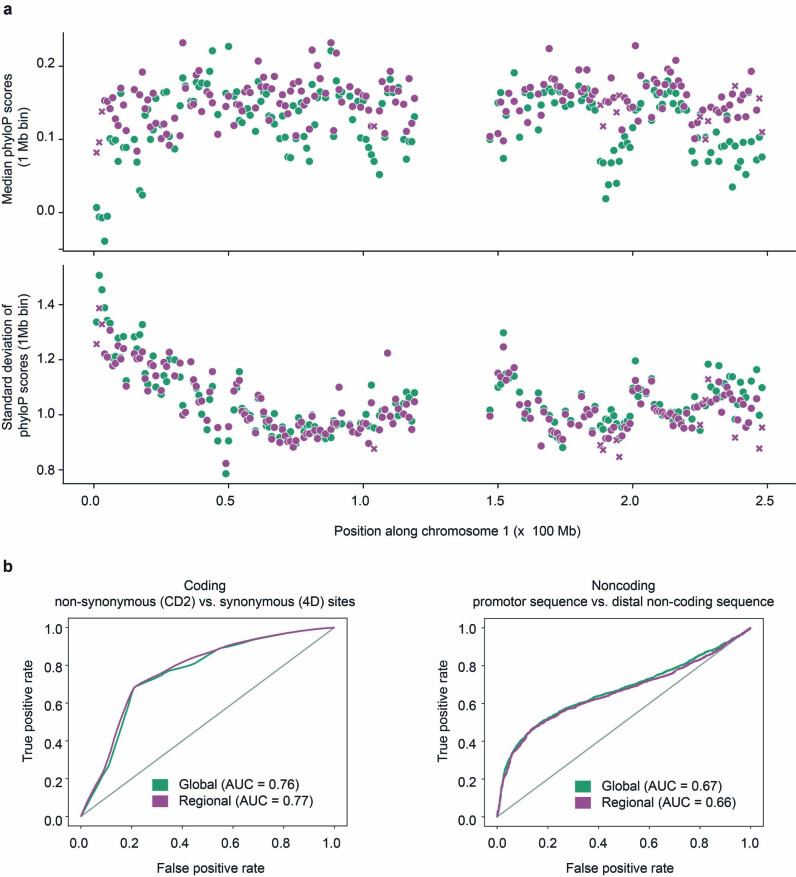
Regional and global substitution models. **(a)** Comparison of neutral background models with genome-wide random sampling of ancestral repeats from all autosomes (green) versus regional modeling of substitution rates at a 1 Mb scale (purple). The upper panel shows median phyloP scores in 1 Mb windows along chromosome 1, the lower panel the corresponding standard deviations. Median scores and dispersion are very similar between global and regional neutral models, values of larger discrepancy tend to fall within windows that containe a limited number of ancestral repeat sequences used to calibrate the regional model, resulting in less reliable estimates of local substitution rates (<50 kb, annotated as purple crosses). **(b)** Comparison of performance of global versus regional model at separating codon position 2 (amino acid-altering positions) versus 4-fold degenerate sites (synonymous positions), and promoters versus matched distal non-coding sequence. Global and regional models achieve similar performance on both coding and non-coding benchmarks.

Expanding the number of available primate species in the MSA to 239 increased the phylogenetic branch length 2.8-fold over the previously available 43 primate species alignment from the Zoonomia study^20^. We used phyloP^26^ to estimate genome-wide per-base constraint for regions of the MSA without ambiguous alignments and found that 3.1% of the bases in the human genome were nominally constrained across all primates (phyloP score > 1.3 or *P* < 0.05), compared with 7.1% of bases that were constrained in the broader set of 240 mammals at the same thresholds. We additionally detected 157 Mb of constrained sequence elements in the primate order using phastCons^26^, comprising 5.1% of the human genome. To determine whether primate constraint metrics could distinguish functional from neutral sequence, we investigated constraint scores in annotated sequence elements. First, we observed that protein-coding DNA—including exons, start codons and stop codons—was strongly enriched in phastCons elements (Fig. 1e). Noncoding DNA encompassing transcribed regions and *cis*-regulatory elements (CREs) in accessible chromatin or occupied by a transcription factor was also significantly enriched. We observed periodic patterns of codon constraint that differentiate exonic from surrounding intronic sequences at the nucleotide level (Fig. 1e). Primate phyloP also distinguished between non-synonymous and fourfold degenerate sites, although less well than mammal phyloP, which is better powered, given the higher total branch length in the mammal MSA (Extended Data Figs. 1 and 2).

**Extended Data Fig. 3:**
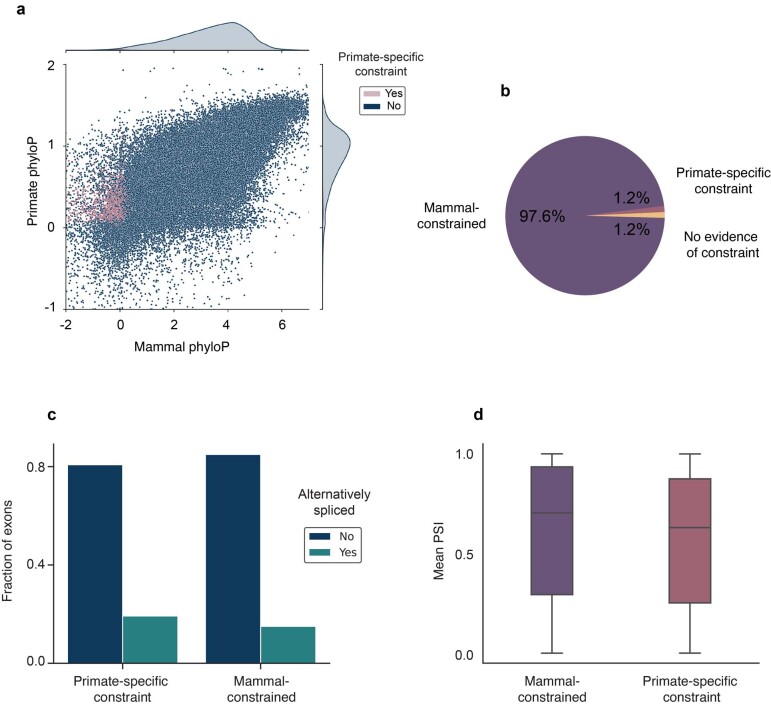
Constraint in human protein-coding exons. **(a)** Average per-base mammal and primate phyloP scores for human canonical protein-coding exons classified by primate-specific constraint. **(b)** Distribution of constraint across clades for 185,275 protein-coding exons. Most human protein coding exons are deeply constrained. **(c)** Fraction of alternatively spliced exons for exons constrained either specifically in primates, or broadly across mammals. Exons with primate-specific constraint are alternatively spliced significantly more often than broadly constrained ones (OR = 1.35, P = 1.3 × 10^−7^, two-sided Fisher’s Exact Test). **(d)** Mean exon inclusion rates (PSI) of alternatively spliced exons across GTEx tissues. Exons constrained specifically in primates have significantly lower inclusion rates than broadly constrained ones (P = 8.6 × 10^−6^, two-sided Rank Sum Test, n = 28,127 exons). Boxes show mean and interquartile range (IQR), whiskers delimit +/− 1.5 x IQR.

We next explored whether we could identify protein-coding genes and exons that are constrained specifically in primates but not in other placental mammals^27^. We estimated primate and non-primate mammal sequence constraint in canonical protein-coding exons annotated in the human genome, identifying 179,329 exons with evidence of constraint in primates at a false discovery rate (FDR) of 5%. As expected, 99% of these exons were broadly constrained across non-primate mammals and vertebrates, but 2,178 were constrained specifically in primates (Extended Data Fig. 3a,b). The majority of primate-constrained exons (72%) are annotated as protein-coding at orthologous regions in the mouse genome, indicating that they are not newly evolved coding sequences but instead have been subject to shifts in selective constraint in the primate order. Genes that had at least one exon constrained among primates but none across other mammals (Supplementary Data 2) were most highly enriched for involvement in the antibacterial humoral response (fold enrichment = 26.4, *P* = 1.8 × 10^−9^; Supplementary Table 2). The overall structure and splicing of these genes were broadly constrained across mammals, suggesting that the amino acid sequences that they encode may have become constrained early on in primate evolution as a maintained response to pathogens. Primate-specific constrained exons were also significantly more likely to undergo alternative splicing (*P* = 1.3 × 10^−7^) and had lower levels of transcript inclusion (*P* = 8.6 × 10^−6^; Extended Data Fig. 3c,d), hinting at an initially limited utilization of recently evolved exons^28–31^. Our results underscore that the evolution of new protein-coding genes or exons from existing sequences is rare, whereas the increased functional importance of pre-existing exons is a relatively more common, but still infrequent, event^32^.

## Primate-constrained CREs

Although comparative genomic and epigenomic studies of mammals and other vertebrates have identified many CREs in the human genome with shared gene-regulatory functions^33,34^, the majority of human DNase I hypersensitivity site (DHS) elements and transcription factor binding or occupancy sites (TFBSs) currently lack detectable sequence constraint^35,36^. This lack of observed constraint in non-primate ancestors might reflect a true divergence in function at these elements, but could also be owing to recently acquired sequence constraint in the primate order^37^.

**Fig. 2:**
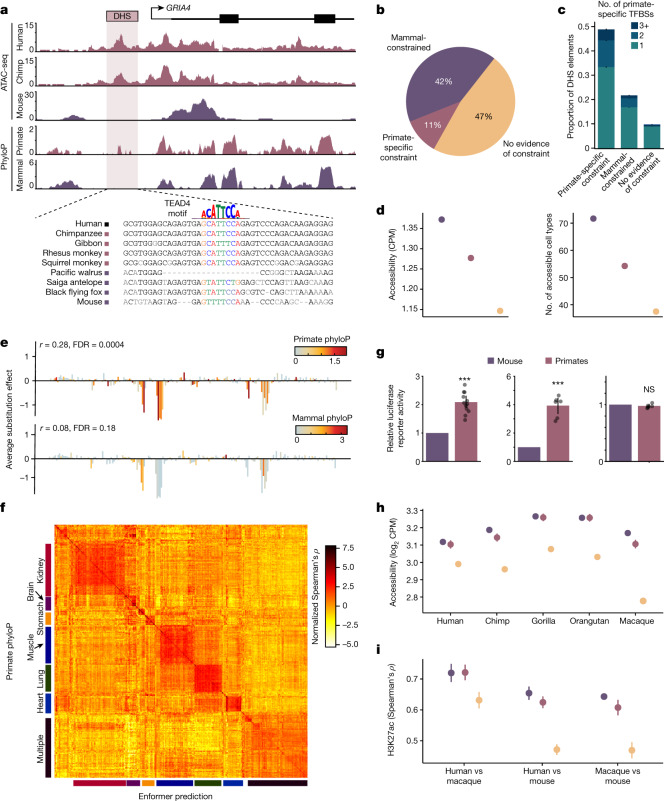
Identification of noncoding regulatory elements with primate-specific constraint. **a**, Example of a primate-specific constrained DHS element in the *GRIA4* locus (hg38; chromosome (chr.) 11:105608279–105612792). Assay for transposase-accessible chromatin with sequencing (ATAC-seq) insertions from human, chimpanzee and mouse iPS cells and phyloP constraint in primates and mammals. A putative *TEAD4* binding motif that better matches primate sequences than non-primate mammal sequences is indicated. **b**, Proportion of constrained DHS elements across clades. **c**, Number of primate-specific constrained footprints (TFBSs) in DHS elements, stratified by constraint across the entire DHS. Error bars represent 95% confidence intervals. **d**, Average chromatin accessibility and the number of accessible cell types is higher at more constrained DHS elements. Colours indicate constraint categories from **b**. Error bars represent 95% confidence intervals. CPM, counts per million. **e**, A saturation mutagenesis experiment (MPRA) of a DHS element at chr. 2:191049304–191045304 (hg38). Average effects of substitutions at each nucleotide on transcriptional activity are correlated with phyloP scores from primates but not from mammals. **f**, Heat map of normalized correlation values (Spearman’s *ρ*) between primate phyloP and sequence-based Enformer predictions of regulatory activity across 438 ENCODE cell types. Categories of similar cell types corresponding to specific tissues are indicated. **g**, Normalized luciferase reporter activity in human iPS cells for three selected sets of primate-specific constrained DHS elements at orthologous primate and mouse sequences. Colours indicate constraint categories from **b**. Bars represent mean and error bars represent 95% confidence intervals; *n* = 36 across 3 elements. *P* values: 1.4 × 10^−5^ (left), 2.8 × 10^−4^ (middle) and 0.54 (right). Raw data are provided in Supplementary Data 6. **h**, Average chromatin accessibility in fibroblasts for five primate species at orthologous sequence elements stratified by sequence constraint. Colours indicate constraint categories from **b**. Error bars represent 95% confidence intervals; *n* = 90,827 DHS elements. **i**, Average Spearman *ρ* of H3K27ac levels at orthologous CREs for three pairs of species. Colours indicate constraint categories from **b**. Error bars represent 95% confidence intervals. *n* = 12 for human versus mouse, *n* = 10 for all other comparisons. ***P < 0.001; NS, not significant.

**Extended Data Fig. 4:**
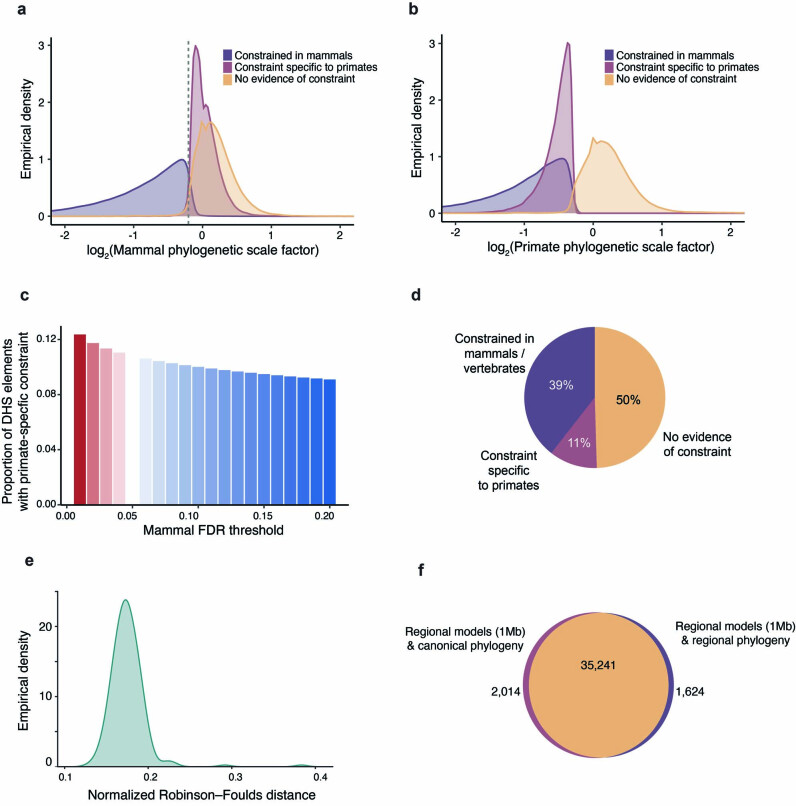
Sensitivity analysis of constraint in DHS elements. **(a)** Distribution of non-primate mammalian scaling factors for DHS elements stratified by clade-specificity of constraint. The dashed gray line denotes where the mammal-constrained and primate-specific constrained distributions intersect. **(b)** Distribution of primate scaling factors for DHS elements stratified by clade-specificity of constraint. **(c)** Proportion of DHS with primate-specific constraint for variable FDR cutoffs in mammals excluding primates. Primate FDR is fixed at 5%. **(d)** Proportion of constrained DHS elements across clades when modeling substitution rates at a 1 Mb scale, compare to Fig. 2b. The estimated proportions are robust to differences between neutral substitution rates modeled in a regional 1 Mb context and a genome-wide averaged model. **(e)** Normalized Robinson–Foulds distance between 1 Mb scale phylogeny and canonical phylogeny along human chromosome 1. **(f)** Venn diagram intersecting DHS elements on chr1 classified as constrained in primates using regional substitution rate models and a fixed, canonical topology, or regional substitution rate models and a variable, regional topology. Models that accounting for regional differences in topology due to e.g. incomplete lineage sorting are highly concordant to those that use a single genome-wide topology (OR = 806.5, P ≈ 0, two-sided Fisher’s Exact Test).

We estimated the average sequence constraint for primates and mammals in high-resolution maps of 1.2 million DHS elements from 438 cell types^8^ (Methods). At an FDR of 5%, we observed that 35% and 33% of elements exhibited evidence of constraint across mammals or within primates, respectively, and largely overlapped (Supplementary Data 3, OR = 14.1, *P* < 1.0 × 10^−300^). After removing DHS elements with ambiguous or contradictory evidence of constraint (Methods), we observed that 42% had evidence of sequence constraint in species that had diverged over 100 million years ago (Ma) (42%), and 111,318 (11%) were significantly constrained in primates but lacked evidence of constraint in mammals or vertebrates (Fig. 2a,b, Extended Data Fig. 4a,b and Methods). The identification of these elements was largely consistent regardless of constraint metric (phyloP or phastCons, OR of overlap = 12.7, *P* < 1.0 × 10^−300^), and sensitivity analyses suggested that the identification of primate-specific DHS elements was robust to mammalian FDR thresholds, regional differences in mutation rates and effects of incomplete lineage sorting (Extended Data Fig. 4c–f).

**Extended Data Fig. 5:**
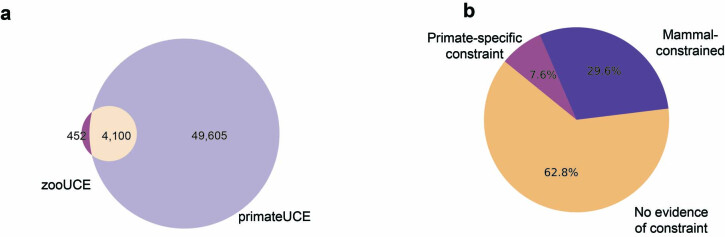
UCEs and constrained TF footprints. **(a)** Overlap between ultraconserved elements as recently defined by Zoonomia (zooUCEs) and primate UCEs allowing up to 1% missing data. **(b)** Distribution of constraint across clades for TF footprints assessed in this study.

Within these DHS elements, transcription factor occupancy prevents DNase I cleavage to create footprints of transcription factor binding events at nucleotide resolution^8,38^. Across 3.6 million TFBS footprints, we find that 1,034,832 (30%) have evidence of broad constraint in mammals, whereas 267,410 (8%) show primate-specific constraint (Extended Data Fig. 5 and Supplementary Data 4). Consistent with previous work, a substantial fraction of footprintable regulatory elements exhibited complex architecture (37%) and contain multiple TFBSs with differing evolutionary constraints on their binding sequences^39^ (Methods). Of note, 66% of DHS elements with primate-specific constraint have a TFBS with evidence of constraint in mammals, suggesting that regulatory function initially evolved in a common ancestor (Fig. 2c). However, 19% of mammal-constrained DHS elements contain individual TFBS footprints with evidence of primate-specific constraint, suggesting that the function of deeply constrained elements can further evolve. Furthermore, we find evidence that the number of DHS elements with primate-specific constraint is likely to be underestimated by phyloP owing to short branch lengths, including 208,717 DHS elements with primate-specific constraint detectable only by phastCons and an additional 86,987 unconstrained DHS elements with at least one primate-specific TFBS. Overall, we find that a significant fraction of putative human CREs have evidence of constraint in primates but not in mammals or vertebrates.

We undertook several studies to validate the biological function of these putative regulatory elements with evidence of constraint specific to the primate order using orthogonal computational and experimental approaches. First, we investigated whether they were more likely to have a regulatory function in humans than elements without detectable constraint. Broadly constrained and primate-specific constrained elements had higher chromatin accessibility and were accessible in significantly more cell types than unconstrained elements (*P* < 1.0 × 10^−300^ for both; Fig. 2d). Across massively parallel reporter assays^40^ (MPRAs) of 148 *cis*-regulatory sequence elements, both mammal and primate constraint at the nucleotide level were significantly correlated with transcriptional changes in saturation mutagenesis experiments (49% and 35%, respectively), of which 14% correlated with primate constraint only (Fig. 2e and Supplementary Data 7). Since elements with primate-specific constraint appeared to have more cell-type-specific biochemical activity than broadly constrained elements, we also tested whether the extent of primate constraint at an element was consistent with cell-type-specific regulatory activity using Enformer^41^, a deep-learning method that predicts gene expression from sequence without using sequence constraint. Across 438 cell types, we observed that primate constraint correlated better with estimates of gene-regulatory activity when the element was accessible in similar cell-type categories to the Enformer predictions (Fig. 2f). Together, these results indicate that regulatory elements with evidence of sequence constraint specific to primates have important *cis*-regulatory functions in humans.

**Extended Data Fig. 6:**
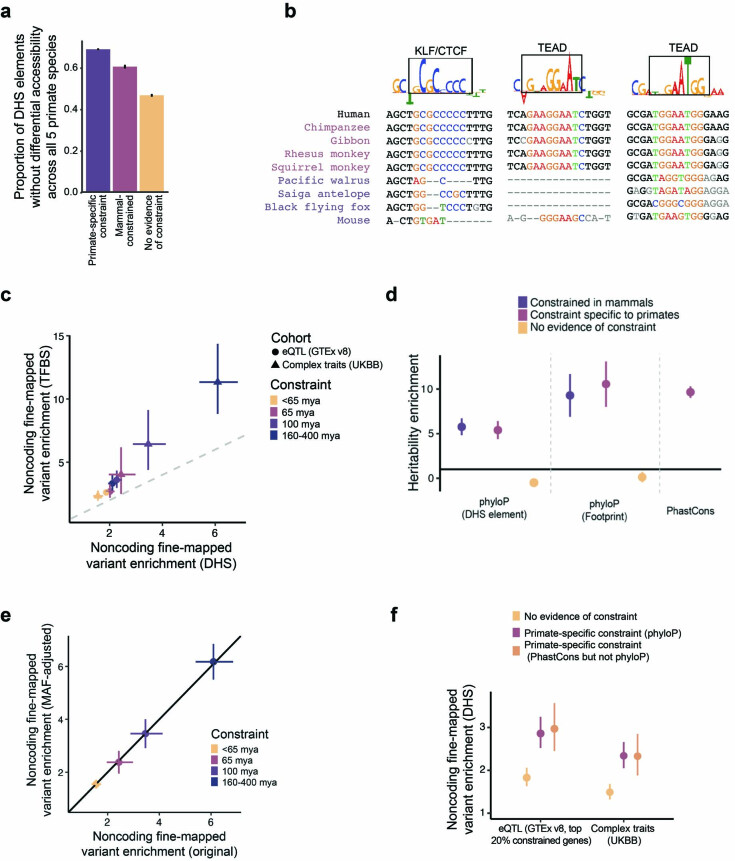
Extended characterization of constrained noncoding regulatory elements. **(a)** Differential chromatin accessibility at orthologous sequence elements across 5 primate species. The y-axis indicates the proportion of elements where differential accessibility was not detected in (37), stratified by sequence constraint. **(b)** For elements tested by Luciferase reporter in Fig. 2g, multiple sequence alignments for select primate and mammal species are shown for a subsequence of tested elements. Subsequences with high DeepLift contribution scores that had matching TF motifs were selected and these data are shown. (**c**) Comparison between the enrichment of fine-mapped variants (PIP > 0.5) in DHS elements or further restricted to TFBSs is shown, related to Fig. 4a,b. Error bars represent 95% CIs, centers represent point estimates. A grey dashed line indicates y = x. The shape of the point indicates whether the enrichment is for eQTLs or complex traits. Colors indicate sequence constraint. n = 3,221 on x-axis and 3,447 on y-axis fine-mapped variants. **(d)** Heritability enrichment as measured by LD Score regression for 6 regulatory constraint annotations and primate Phastcons. n = 69 traits. Error bars represent 95% CIs. **(e)** Comparison of noncoding fine-mapped variant enrichment with and without adjustment for MAF distributions between the set of variants with PIP > 0.5 and the set with PIP < 0.01. Error bars represent 95% CIs, centers represent point estimates. n = 3,221 fine-mapped variants. **(f)** Enrichment of fine-mapped variants (PIP > 0.5) in DHS elements, related to Fig. 4a,b. Error bars represent 95% CIs, centers represent point estimates. Colors indicate sequence constraint, including primate specific constraint as defined by phyloP and by phastCons but not phyloP. n = 3,221 for UKBB and 48,183 for GTEx fine-mapped variants.

In addition to the extensive body of human experimental data providing support for the function of primate-constrained regulatory elements, a limited number of experiments have been conducted in non-human primates, enabling us to investigate the regulatory activity of primate-constrained DHS elements in non-human contexts. First, we set out to experimentally validate the regulatory capacity of a small subset of DHS elements with primate-specific constraint. We cloned orthologous sequences from human, chimpanzee and mouse into luciferase reporter assays, transfected these constructs into human induced pluripotent stem cells (iPS cells), and measured transcription of the reporter gene for three elements. Of note, two out of three elements drove transcription more strongly from the primate sequences than from the mouse sequence (Fig. 2g and Supplementary Data 6), and we set out to validate this observation more broadly. We investigated chromatin accessibility across DHS elements in fibroblasts from four non-human primate species, observing that primate-specific constrained DHS elements displayed higher and more consistent chromatin accessibility in all four primate species compared to unconstrained DHS elements^42^ (Fig. 2h and Extended Data Fig. 6a). We also investigated the levels of H3K27ac, a marker of active CREs, in stage-matched cell types during corticogenesis at orthologous regions in humans, rhesus macaques and mice^43^. We observed that H3K27ac levels at deeply constrained and primate-specific constrained elements were significantly better correlated between human and macaques than at elements without evidence of constraint (*P* = 0.0004 and 0.0001, respectively; Fig. 2i), indicating that constraint on the sequence level corresponds to constraint of molecular function between species. Nevertheless, primate-specific constrained elements also shared functional similarity between primates and mouse, consistent with the results of our TFBS analyses.

**Fig. 3:**
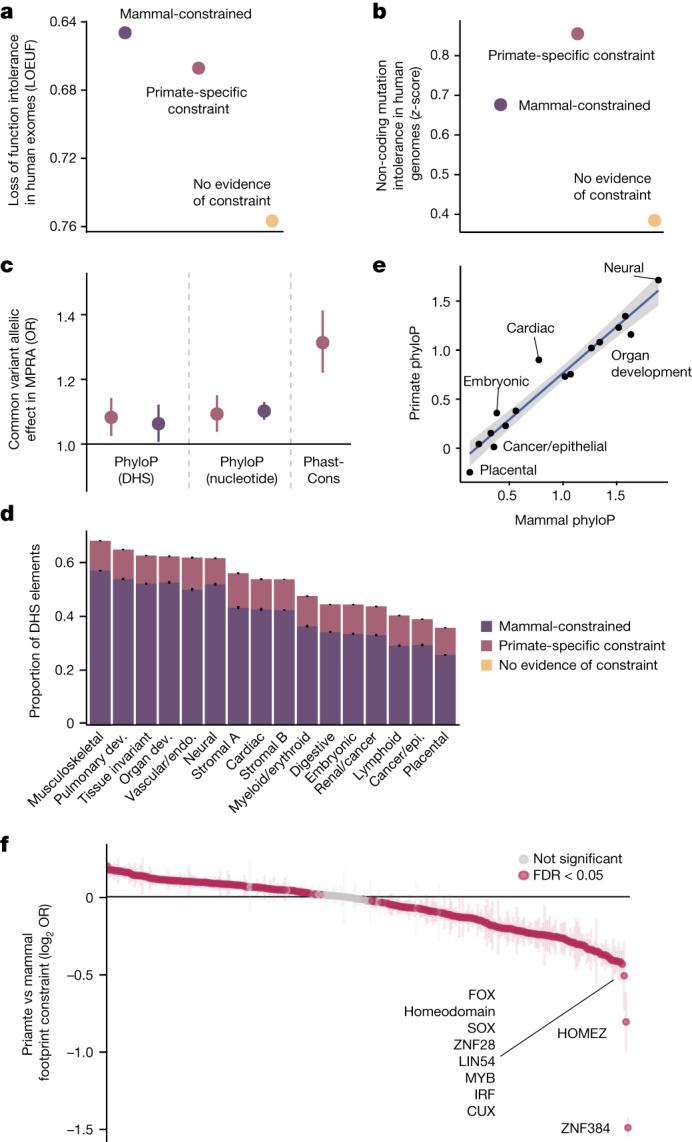
Characterization of constrained regulatory elements. **a**, Predicted target genes have fewer loss-of-function mutations in humans than expected at constrained DHS elements. Error bars represent 95% confidence intervals. **b**, Constrained DHS elements have fewer mutations in human populations than unconstrained elements. Error bars represent 95% confidence intervals. **c**, Enrichment of allele-specific regulatory activity (MPRA) for 27,023 common variants, stratified by type of constraint. A colour legend for constraint categories is shown in **d**. Error bars represent 95% confidence intervals, the central dot represents point estimates; *n* = 27,023 variants. **d**, Proportion of constrained DHS elements across 16 broad cellular contexts. Error bars represent 95% confidence intervals, centre represents proportion. *n* = 1,029,688 DHS elements. Dev., development; endo., endothelial; epi., epithelial. **e**, Scatter plot of mean primate and mammal phyloP scores at DHS elements, stratified by cell types. A linear fit is shown with a corresponding 95% confidence interval ribbon. Putative outlier cell types with higher primate phyloP than mammal phyloP scores are indicated. **f**, Differences in the proportion of primate and mammalian constrained footprints in human DHS elements, for each of 283 transcription factor family motifs. Positive values indicate a higher proportion of constrained TFBSs in primates, negative values indicate a lower proportion of constrained TFBSs in primates. Transcription factors that are the least constrained in primates compared to mammals are labelled, and significantly different transcription factors are coloured in magenta (FDR < 5%). Error bars represent 95% confidence intervals.

Evolutionary constraint estimated in mammals and vertebrates is correlated with selective constraint estimated in human populations^17,44^, so we explored contemporary human cohorts for evidence of ongoing selection against genetic variants that disrupt primate-constrained regulatory elements. Using the gnomAD cohort of 141,456 human individuals^45^, we found that predicted target genes of primate-specific elements had significantly fewer loss-of-function mutations than expected (*P* < 10^−300^; Fig. 3a). Moreover, we observed increased mutational constraint^46^ in the noncoding primate-specific constrained elements themselves (*P* < 10^−300^; Fig. 3b). Indeed, polymorphic variants in regulatory elements were more likely to have allele-specific regulatory effects by MPRA when there was evidence of constraint in primates at the mutated nucleotide (*P* = 0.0007) or across the entire regulatory element (*P* = 2.9 × 10^−13^; Fig. 3c), even after controlling for mammalian constraint (*P* = 1.1 × 10^−5^). Together, these results extend previous studies^44,46^ and suggest that regulatory elements constrained specifically in the primate order are under purifying selection in human populations and that mutations in these elements are likely to have important regulatory functions.

To explore whether genes expressed in specific tissues were more likely to be regulated by noncoding elements with primate-specific constraint, we investigated the depth of conservation across 16 broadly defined cellular contexts^47^. We confirmed that regulatory elements active in multiple cell types—and particularly in neural and musculoskeletal cell types—were most deeply constrained^48^, whereas blood, epithelial, and placental cell types were least constrained (Fig. 3d). Regulatory elements present in neural, cardiac and embryonic cell types exhibited higher phyloP scores in primates than in mammals (Fig. 3e). We explore the connection between ultraconserved elements (UCEs) and neural cell types below. Finally, we investigated whether specific TFBSs were more or less constrained in primates than in mammals, finding that most TFBS motifs in DHS footprints had significant, but small, differences (241 out of 282 (85%); Fig. 3f). A small number of footprints are over 20% less constrained in primates than mammals, including the KRAB zinc-finger domain transcription factors (KZNFs), ZNF384 and ZNF28. The reduced constraint at KZNF binding sites in primates probably reflects the divergence of KZNFs themselves, which are among the fastest evolving gene families in primates^49,50^.

## Ultraconserved elements in primates

In addition to the elements that we detected as constrained by phyloP and phastCons, we identified 74.6 million positions in the human genome that are perfectly conserved without a single substitution across all 239 primate species. These positions were often contiguous, and we catalogued 33,368 primate UCEs that were at least 20 bps in length (Supplementary Data 5), amounting to more than 1 Mb of total DNA sequence including 7,261 coding exons and 22,582 DHS elements. More than half (57%) of the 4,552 recently described mammalian UCEs^18^ overlapped our primate UCEs, and 82% overlapped after allowing for up to 1% of missing species per aligned column within the primate alignment. Genes whose protein-coding sequences overlapped primate UCEs were more likely to be involved in nervous system development (Supplementary Table 3, fold enrichment = 2.24, *P* = 8.8 × 10^−9^). We additionally found that 2.7% of primate UCEs also overlapped brain regulatory elements (fold enrichment = 3.1, *P* < 10^−300^), consistent with the deep constraint of neuronal protein-coding sequences.

## Complex trait variation in constrained CREs

**Fig. 4:**
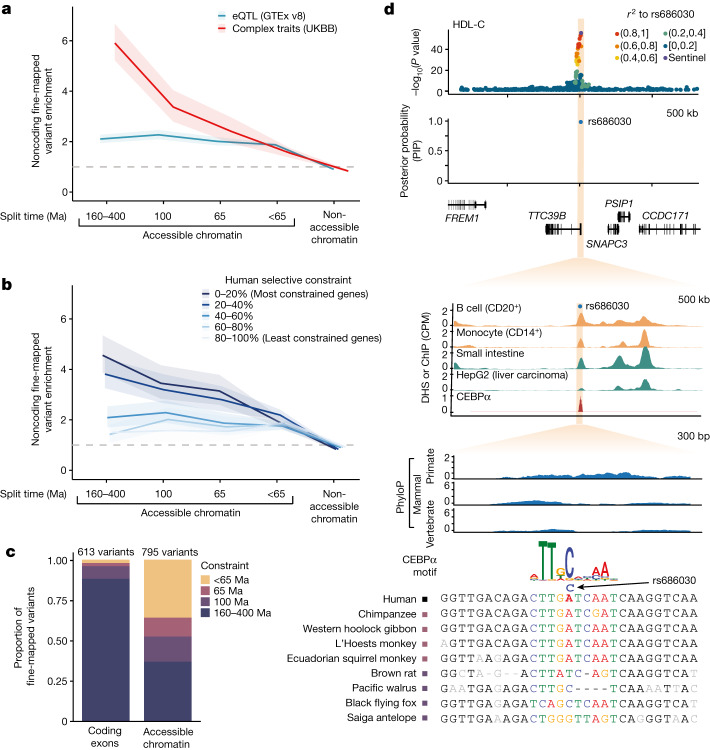
Enrichment of complex trait variants at constrained noncoding CREs. **a**, Enrichment of fine-mapped GWAS variants from 96 UK Biobank (UKBB) complex traits and clinical phenotypes (red) or eQTLs for 49 GTEx tissues (blue) in DHS elements, stratified by sequence constraint of the element. Approximate split times for vertebrates (160–400 Ma), placental mammals (100 Ma) and primates (65 Ma) are shown. Enrichments are computed as the ratio of the proportion of variants with PIP > 0.5 compared to the proportion of variants with PIP < 0.01. Ribbons represent 95% confidence intervals and the centre represents the point estimate. The grey dashed line indicates an OR of 1. **b**, Enrichment of fine-mapped eQTL variants within DHS elements as in **a**, with genes separated into five bins based on their selective population constraint (LOEUF). Ribbons represent 95% confidence intervals and the centre represents the point estimate. **c**, Total count of fine-mapped variants for 96 UK Biobank phenotypes in protein-coding exons or accessible chromatin sites, stratified by extent of constraint as in **a**. **d**, Example of a fine-mapped variant (rs686030) for HDL-C and cholelithiasis at a primate-specific constrained DHS element. GWAS signal at the locus, fine-mapping probability, DNase signal, CEBPα chromatin immunoprecipitation with sequencing (ChIP–seq) signal, constraint scores and MSAs of primate (blue) and mammal (green) species are shown.

Genome-wide association studies (GWAS) have identified hundreds of thousands of genetic variants associated with complex human diseases and changes in gene expression, the majority of which map to noncoding CREs^27,33,34,37^. We identified DHS elements and footprints containing fine-mapped GWAS variants (posterior inclusion probability (PIP) > 0.5) for 96 human clinical phenotypes and complex traits from the UK Biobank^8,47^, and characterized whether the underlying sequence was constrained only in primates (65 Ma), placental mammals (100 Ma), vertebrates (160–400 Ma), or without evidence of constraint (less than 65 Ma; Fig. 4a and Extended Data Fig. 6c). Fine-mapped variants underlying clinical phenotypes and complex traits were enriched across all classes of distal accessible chromatin element and footprints, including those with primate-specific constraint (OR = 2.4, *P* = 2.5 × 10^−13^ and OR = 4.0, *P* = 1.8 × 10^−7^, respectively), with more deeply constrained elements showing greater enrichment^51^. A heritability enrichment analysis corroborated the relevance of constrained regulatory elements and primate-specific constraint more generally in complex traits (Extended Data Fig. 6d). In comparison, fine-mapped variants underlying changes in gene expression (expression quantitative trait loci (eQTLs)) from the GTEx study showed similar enrichment for elements with recent constraint but were markedly less enriched at elements that are broadly constrained across mammals or vertebrates. After stratifying human genes by selective constraint quantified by loss-of-function observed/expected upper bound fraction (LOEUF) scores^38^, we found that variants affecting the expression of highly constrained genes tended to be enriched at more deeply constrained DHS elements and footprints (OR = 4.6, *P* = 1.0 × 10^−53^ and OR = 8.0, *P* = 4.3 × 10^−24^, respectively), whereas variants affecting the expression of less constrained genes tended to reside at elements with more recent constraint (Fig. 4b).

To explore the functional role of primate-specific constrained CREs in human complex traits and clinical phenotypes, we partitioned the fine-mapped variants from the UK Biobank by protein-coding consequence and constraint depth. In contrast to 88% of fine-mapped protein-coding variants residing within deeply constrained exons that predate the emergence of placental mammals (Fig. 4c and Supplementary Data 8), only 37% of noncoding variants in accessible chromatin were constrained to this extent. 12% of fine-mapped variants in CREs were constrained only in primates and not in placental mammals, corresponding to 93 probably causal regulatory variants underlying human complex traits and clinical phenotypes (Supplementary Data 9 and 10). One example is rs686030, a fine-mapped noncoding variant in a primate-constrained DHS element near the TCC39B gene, which is associated with high-density lipoprotein (HDL) cholesterol levels (PIP = 0.99) and cholelithiasis (PIP = 0.38) (Fig. 4d). The derived allele strengthens a motif for the bound CEBPα transcription factor and is associated with *TCC39B* gene expression (PIP = 0.43 for liver), and mouse knockout studies of *TTC39B* showed an increase in HDL-C levels^52^, potentially modulating the risk of cholelithiasis via bile cholesterol secretion. Although 36% of fine-mapped variants at DHS elements lack significant constraint across primates and other mammals, these elements were also not significantly enriched for heritability in humans (Extended Data Fig. 6d), suggesting that further data are needed to resolve these loci, some of which might be false positives^53^. Of note, we find residual enrichment for fine-mapped variants in DHS elements that lack evidence of constraint by phyloP (FDR < 5%) but overlap with phastCons elements in primates (Extended Data Fig. 6f). Additional sequencing to increase sampling density on this branch may help to define the selective constraints at the origin of our own species and their contribution to human clinical phenotypes and complex diseases.

## Discussion

Heritable modifications in genomic sequence are necessary for trait adaptations and the emergence of new species, but the nature of these sequence changes remains incompletely understood. Although constrained noncoding elements in mammals have been extensively catalogued, less attention has been paid to those in the primate lineages, in part owing to the challenges in detecting constraint at short phylogenetic distances with previously available species sampling. By placing the genomes of 239 primate species, including 187 newly assembled here, in the context of other mammalian and vertebrate genomes^20^, we identified hundreds of thousands of constrained noncoding sequence elements and catalogued the origins of their sequence constraint in primates, placental mammals and more distant vertebrates. Collectively, these CREs are unique evolutionary records that provide a lens through which to view the mechanisms of recent exaptations leading to our species^10^.

In keeping with prior work showing that noncoding DNA evolves more rapidly than protein-coding sequences^17,18,54,55^, we find that many human CREs that previously showed no evidence of sequence constraint are in fact constrained exclusively in primates, considerably expanding the number of known constrained noncoding elements in the human genome. Indeed, sequence constraint in primates uniquely predicted the function of a subset of regulatory elements, and specifically constrained elements had higher and more similar regulatory functions in diverse human cell types and across distinct primate species. These elements are predicted to regulate genes that are more intolerant to deleterious mutations in human populations and are significantly enriched for common genetic variants associated with variation in gene expression and complex human traits and diseases. Nevertheless, some functional genomic elements underlying complex human phenotypes do not show evidence of constraint in either primates or mammals in our analysis, suggesting that they potentially emerged after the initial radiation of primates and thus became selectively constrained only in a sub-lineage such as anthropoids or apes, or that functional sequence elements were selectively lost in one or more lineages. Additional sequencing of the remaining species in the primate order, including population-level oversampling of key lineages, would help to provide the resolution needed to detect sequence elements under selective constraint in finer detail, especially those specific to clades from which the human species ultimately emerged.

## Methods

### De novo assembly and repeat-masking

To maximize the species diversity of primates in our analyses, we newly sequenced and assembled the genomes of 187 different primate species, initially presented in refs. ^11,23^, for which no other reference genome assembly was available. In brief, each individual was sequenced with 150 bp paired end reads on the Illumina NovaSeq 6000 platform to an average whole-genome coverage of ~35×, and we assembled the resulting reads into contigs using Megahit^25^ (version 1.2.9) using default parameters. The resulting assemblies had an average contig N50 of 34 kb, and the assembly sizes ranged from 2.1–3.0 Gb, thus falling within the typical range of previously reported genome sizes for primates^56^ (see Extended Data Fig. 1a). We then combined these assemblies with the reference genomes of 52 additional species that had been previously generated as part of other studies^57^ and or available through public repositories (Supplementary Data 1). The final species sampling densely covers the whole primate radiation and includes members of all 16 primate families and 72 primate genera. We identified and soft-masked common genomic repeats within the assemblies using RepeatMasker (version 4.1.2-p1; http://www.repeatmasker.org), utilizing the primates repeat catalogue as query.

### Multiple sequence alignment

We aligned the assemblies with Cactus^21^ (version 2.1.1), using the phylogeny presented in^11^ as a guide tree for progressive decomposition, and used the previously available high-quality assemblies as alignment outgroups. All computation was done by running cactus-prepare with options–wdl–noLocalInputs–preprocessBatchSize 5–defaultDisk 3000 G–halAppendDisk 9000 G–defaultCores 64–gpu–gpuCount 8–defaultMemory 385 G–alignMemory 450 to produce a script in workflow description language (WDL), then uploading it to Terra (https://app.terra.bio/) where it was executed on Google Cloud Platform. GPU-related issues prevented that version of Cactus from executing to completion, so the job was resumed using a WDL made without the–gpu and–gpuCount options. An outgroup to primates (*Mus musculus* reference mm10) was manually added to the root alignment job by editing the WDL, and the ‘LOCAL’ disk parameter of the hal_append_subtree task was manually increased to 9,000. Cactus has since been fixed (v2.2.3) to resolve all issues encountered during this alignment.

We then combined our resulting primate MSA with the recently generated mammalian MSA by the Zoonomia consortium^20^. In brief, we used hal2fasta from the haltools^21^ package to output the ancestral genome at the root of the primate MSA, and used it to generate a bridge alignment with the Sunda colugo (*Galeopterus variegatus*), the closest outgroup to primates in the Zoonomia MSA. We used this bridge alignment to insert the primate MSA into the Zoonomia MSA, and replace the original primate branch with it.

To generate the final, filtered alignment used as input for subsequent analyses described below, we output maf files centred on the human genome reference using haltools including the “–onlyOrthologs–noAncestors –noDupes” flags, thus removing any regions with potentially ambiguous mappings at multiple locations.

### Pairwise alignments error rate estimate

To quantify residual error rates within the genome assemblies generated in this project, we identified 25 species for which a reference genome was previously assembled with an orthogonal, state of the art combination of technologies (Supplementary Table 1). After introducing a minimum contig length cutoff of 1 kb, we generated pairwise alignments between the two assemblies using minimap2^58^ (v. 2.17-r941) using the following flags:–cs -x asm5. We called variants on the resulting alignments by retaining alignment blocks of at least 1 kb within the PAF file using paftools.js, by applying the following flags: paftools.js call -l 1000 -L 1000. We quantified mismatch rates from the resulting output accounting for the fraction of the genome within alignment blocks, resulting in mismatch rates that range from 0.00026–0.00515 mismatches per bp. As the genome assemblies produced herein are haploid compressions of diploid organisms, a random allele will be sampled and incorporated at heterozygous positions, and thus the resulting differences between two assemblies of the same species should be strongly correlated with the species’ intraspecific diversity. We compared our mismatch rates to the estimates of heterozygosity for the same genomes presented in ref. ^11^, and confirmed that heterozygosity accounts for 83% of the observed variation in mismatch rates across assemblies. We quantified the residual mismatch rate after regressing out it’s the effects of heterozygosity, and found the resulting average mismatch rate to be 0.0004 mismatches per bp, which we consider to be sufficiently low for our analyses. We note that the number of base differences due to assembly error is likely lower than this, as residual mismatches also include fixed differences between individuals, which are not accounted for by heterozygosity.

### Detecting selective constraint

We measured selective constraint genome wide using the widely used phyloP and phastCons algorithms from the PHAST package^26,59^. To do so, we extracted the ancestral genomes of primates and of eutherian mammals from our alignment using haltools hal2fasta, and annotated common genomic repeats in both using ReapeatMasker as described above, but using the mammalian repeat catalogue for the eutherian ancestor. We lifted the resulting annotations into human reference space, and randomly sampled 1 Mb of autosomal short interspersed nuclear element (SINE), long interspersed nuclear element (LINE), long terminal repeat (LTR) and DNA repeats from the alignments as putatively neutrally evolving regions. We used these regions as input for phyloFit together with the general reversible model (“–subst-mod REV”) as the nucleotide substitution model and expectation maximization algorithm (“-EM”) to fit it to the data. As our goal is to detect elements with sequence constraint specific to primates, we generated the neutral background models once for all primates, and once for all mammals after excluding the primate branch. We additionally generated a neutral model for the 100-way vertebrate MSA from UCSC Genome Browser in our analysis to minimize false negatives on the mammalian track, for which we also excluded the primate branch containing 11 species and defined neutral background models via alignments at 4D sites as putatively neutral regions, due to their easier detection across the much larger phylogenetic distances present in this alignment.

We used the models to estimate constraint in different ways across the three clades (primates, mammals, vertebrates): For phyloP, we calculated scores for both constraint and acceleration with the “–mode CONACC” flag, and used the likelihood ratio test “–method LRT” yielding phyloP scores—that is, the −log_10_(*P* value) from the hypothesis test, and the associated scale factor. We scored individual bases by outputting them via the “–wig-scores” flags. We additionally scored element-wide annotations for coding sequences, DHS and TFBS by passing them to phyloP via the “–features” flag, to increase power as the test is performed across more than a single basepair. Finally, we generated discrete constrained elements in primates using phastCons, using primate neutral background model, the “–expected-length 45–target-coverage 0.3–rho 0.31” consistent with previous studies^18^, and output constrained elements with the “–most-conserved” flag.

To explore the potential impact of regional variation in substitution rates on our estimates of constraint, we additionally generated regional neutral background models for primates and other mammals from 1-Mb sliding windows across the human genome. In each window, we subset the previously identified ancestral repeats and randomly selected 100 kb of sequence after trimming sites with >20% missing data. As described above, these sites were used to estimate substitution rates input with phyloFit, and the resulting models were used to run phyloP for individual bases and DHSs elements.

To additionally ensure our estimates of constraint are robust to topological variation in the underlying phylogeny due to potential sources of uncertainty such as incomplete lineage sorting, we additionally inferred regional phylogenies for primates using a maximum likelihood approach implemented in IQtree. In brief, we randomly subset 150 kb of trimmed sequence from each 1 Mb window, which was used to estimate an appropriate substitution model and infer the phylogeny including 1,000 bootstraps. We used the topology of the resulting consensus tree and the ancestral repeat alignments to infer neutral models as described, using the same subset of sites as for the regional models to minimize additional sources of variation, and assessed the concordance of constraint for DHS elements between regional models using the canonical and regional phylogenies.

### Protein-coding exons

To identify protein-coding exons with constrained specifically in the primate lineage, we used phyloP with protein-coding exons from GENCODE (v 42)^9,27^ as element-wise input as described above across the primate, mammalian, and vertebrate tracks. We restricted these analyses to exons that are part of ‘Ensembl canonical’ transcript, and additionally excluded any exon that overlaps known human segmental duplications, as defined by the segmental duplication track on UCSC Genome Browser. We ran element-wise phyloP tests on these remaining coding exons, and defined constrained exons for each clade (primates, mammals, vertebrates) directly based on the resulting *P* values. We accounted for multiple testing by retaining those that remained significant at a 5% FDR^60^. To define exons with primate-specific constraint, we required them to be significantly constrained in primates, but not in mammals or vertebrates. To detect whether these exons also have coding potential in other mammals, we lifted the underlying coordinates to the mouse genomes (mm10) and checked weather they overlap protein-coding annotations there. To define genes with primate-specific constraint, we looked for genes containing one or more exons with primate-specific constraint, but no mammal differentially constrained ones. To calculate differences in the proportion of alternatively spliced exons between broadly constrained and primate specifically constrained exons, we calculated the mean exon inclusion rate across tissues from the GTEx project^61^, and defined exons with an inclusion rate different from 1 as alternatively spliced. A list of exons and genes with primate-specific constraint is presented in Supplementary Data 2.

### GO-term enrichment

We used Panther^62^ to calculate Gene Ontology (GO)-term enrichments of genes with primate-specific constraint, and those overlapping primate UCEs. We used Fishers’ exact to test for statistical overrepresentation on the ‘GO biological process’ annotation, by using the Ensembl identifiers of the underlying genes from either analysis as foreground set and the human gene annotation as background. To account for multiple testing, we report only results that remain significance at a FDR (Benjamini–Hochberg) of 5%.

### DHSs and TFBSs

We obtained high-resolution maps of DHSs from 733 human biosamples encompassing 438 cell and tissue types and states^47^. The study reported 3.6 million DHS elements, and we applied several additional quality control steps to remove low-quality peaks. First, we excluded all peaks without 1-to-1 matches between GRCh38 and hg19. We normalized peaks to 300 bps in size for all analyses, except for the element-wise constraint scoring described below. Finally, we required all peaks to be within the top 100,000 in at least one annotated cell type in the datasets, by the normalized score provided from the study. After excluding sex chromosomes, this resulted in a set of 1,238,405 peaks that were used in downstream analyses. We similarly obtained 3,622,316 consensus DNase I hypersensitivity footprints for the set of DHS elements used in our primary analyses^38^. Cell types and tissues where each DHS element was most strongly associated were previously estimated using non-negative matrix factorization with 16 components^47^.

We defined a core 40-bp window surrounding the summit of the peak of each DHS annotation as the input to calculate element-wise. Analogous to protein-coding exons, we then calculated constraint in DHS and TFBS element-wise using phyloP across primates, mammals, and vertebrates, and define constrained elements in each clade as those remaining significant at a 5% FDR^60^. To define primate-specific constraint in DHS and TFBS, we required the elements to be significantly constrained in primates, but not in mammals or vertebrates. Finally, DHS elements and TFBSs that did not have primate-specific constraint by phyloP but overlapped with a primate PhastCons elements were excluded from the primary analyses for consistency in interpretation, since these sequences represent a mixture of primate-specific and deeply constrained sequences. The depth of constraint for each DHS and TFBS are provided in Supplementary Data 9 and 10. Approximate target genes of each DHS element were based on the closest gene using the ‘nearest’ function the R GenomicRanges package.

### TFBS enrichment analysis

We obtained archetypal motifs overlapping each TFBS or DHS footprint from the annotations presented in ref. ^38^. Footprints typically had multiple motif matches and were considered independently. For each motif, we computed the proportion of footprints in either constraint category (primate or mammal constrained below an FDR of 5%, as described above), where the denominator was the total number of constrained footprints (primate or mammal) regardless of motif match. We then calculated the odds ratio for each motif to test whether the proportion of primate-constrained and mammal-constrained footprints were different. After observing a small bias where short footprints were more likely to be detected as constrained in mammals, we split footprints into 10 equal-sized bins, computed the odds ratio for each motif in each bin, then performed a fixed effects meta-analysis for each motif.

### Primate UCEs

We defined UCEs across primates analogous to ref. ^18^: We filtered regions with ambiguous or multiple alignments using haltools including the “–onlyOrthologs–noAncestors –noDupes” flags, and parsed the resulting alignment to exclude any alignment column that is different from all other species in at least one species. We then kept consecutive stretches of 20 bp or more for the final set of UCEs. For a more lax definition, we allowed for missing data (“-” or “N”) in the alignment in at most 2 species (1%). We strictly defined overlap to previous annotations as 1 bp or more.

### Estimates of constraint in human populations

Gene constraint in the human population was estimated using the LOEUF metric. In brief, this metric conservatively estimates the selection against loss-of-function mutations by taking the upper bound of a 95% Poisson confidence interval around the observed to expected ratio of loss-of-function mutations. LOEUF values were obtained from 141,456 individuals in gnomAD v2^45^. Constraint across noncoding regions of the genome was estimated as a *z*-score for depletion of mutations compared to expectation^46^. *Z*-scores for non-overlapping 1,000-bp bins were obtained from 71,156 individuals in gnomAD v3. When a DHS element overlapped multiple bins the average *z*-score was used.

### Trait-associated variant analyses

Fine-mapping results for 96 complex traits and diseases across 366,194 unrelated ‘white British’ individuals in the UKBB^63^ were obtained from https://www.finucanelab.org/data and have previously been described in detail^64^. In brief, fine mapping was performed using FINEMAP^65,66^ and SuSiE^67^ with GWAS summary statistics from SAIGE/BOLT-LMM and in-sample dosage linkage disequilibrium (LD) computed by LDstore 2^68^. Regions were defined by expanding ±1.5 Mb for each lead variant and were merged if they overlapped. Up to 10 causal variants were allowed per region. Posterior inclusion probabilities (PIPs) were averaged across the two methods and variants where PIPs from the two methods disagreed by >0.05 were excluded.

Fine-mapping results for expression quantitative traits in 49 tissues across 838 individuals were obtained from https://www.finucanelab.org/data and have been described in detail^61,64^. In brief, fine mapping was performed using SuSiE on *cis*-eQTL summary statistics from the GTEx portal (https://gtexportal.org/). Covariates (sex, PCR amplification, sequencing platform, genotype principal components, and probabilistic estimation of expression residuals factors^69^) were projected out from the genotypes prior to fine mapping. After fine mapping, all variants were lifted over from GRCh38 to hg19.

Definition of constraint at DHS and TFBSs was slightly modified such that evidence of constraint out to mammals or vertebrates was separated and elements with discrepant estimates of constraint were excluded. Specifically, constraint at approximately 100 Ma required that mammal and primate phyloP scores were below the FDR threshold but vertebrate phyloP was above the FDR threshold. Similarly, constraint at approximately 160–400 Ma required that vertebrate, mammal, and primate phyloP scores were below the FDR threshold.

Bigwig files for accessible chromatin and transcription factor occupancy were obtained from the ENCODE project^47,70^ (ENCFF220IWU, ENCFF659BVQ, ENCFF619LIB and ENCFF842XRQ) or the Sequence Read Archive (SRX097095). Coding variants were annotated as loss-of-function, missense, or synonymous using the Ensembl Variant Effect Predictor (VEP) v85^71^. When a variant had multiple coding annotations, the most severe consequence on the canonical transcript (GENCODE v19) was used.

We computed the enrichment of fine-mapped variants for different annotations by comparing the proportion of variants with PIP > 0.5 to the proportion of variants with PIP < 0.01. Distal elements were defined as DHS elements that did not overlap promoters^72^. When variants were fine-mapped across multiple traits, tissues, or genes, only the highest PIP variant was used. Confidence intervals and *P* values were estimated using Fisher’s exact test. Enrichments were performed in hg19 and annotations were lifted over from GRCh38.

A similar enrichment analysis was performed using stratified LD score regression (S-LDSC)^72^ to estimate the heritability in each annotation. Similar to previous studies^7^, S-LDSC models were fit using approximately 10 million common variants including the Baseline v2.2 annotations. Annotations derived in GrCH38 were lifted over to hg19, and their LD scores were estimated using the EUR sub-population of the 1000 Genomes project. Enrichment and average per-SNP heritability estimates were meta-analysed across 69 mostly independent traits using a random effects model.

The predicted effects of fine-mapped variants on transcription factor binding was estimated using motifbreakR^73^ for 426 position weight matrices from HOCOMOCOv11^74^. A motif match was determined using the information content (ic) if either allele obtained a *P* value < 0.0001. A variant disrupted a motif match if there was a difference of >0.4 for the scaled motif matrix between alleles.

### Enformer analysis

We obtained the 733 bio-sample aggregated DNase peak dataset as curated by^47^ and deduplicated the technical replicates by retaining the top bio-sample for samples with technical replicates. We retained all DHS peaks found in more than two biosamples for downstream analysis, calculated the midpoint for each DHS and scored the regions using the Enformer model^41^. To assess the local functional relevance of the Enformer scores, we averaged them across ±128 bp around the midpoint of each DHS. To compute the correlation between the Enformer score and phyloP in each bio-sample, we pairwise intersected DHS with primate-specific constraint for all bio-sample pairs, and computed the correlation between the Enformer and phyloP scores for the retained regions, and row and column normalized the final correlation matrix. The final matrix was hierarchically clustered on the rows, and the same order was retained for the columns in the heat map. Major cell types for each correlation block identified are highlighted as annotations.

### Luciferase reporter vector construction

Mouse, chimp and human CRE with 150 bp in length were synthesized by IDT. The CRE was cloned into the linearized pGL3- Promoter vector (cut by Nhel and BglII). The fusion product (pGL3-cRE) was subsequently transformed into Mix & Go Competent Cells Strain Zymo 5-a (Zymo Research, T3007). Clones were selected by ampicillin and plasmids were prepared using the NucleoSpin Plasmid Transfection-grade (Takara, 740490).

### Transfection and luciferase assays

Human iPS cells were transfected in a 24-well plate using the Lipofectamine Stem Transfection Reagent (Invitrogen, STEM00001) and Opti-MEM Reduced Serum medium (Invitrogen, 31-985- 070). On the day of transfection, cell density was 50% confluent. For each well, 500 ng of pGL3-enhancer, pGL3-control, or pGL3-promoter was co-transfected with 10 ng of pRL-CMV (Promega, E2261) as an internal control for the normalization of luciferase activity. Cells were incubated with DNA–lipid complex overnight and media was changed for another two days. The firefly and *Renilla* luciferase activity were measured respectively using a Dual-Glo Luciferase Assay System (Promega, E2920). Human iPS cells were obtained from the Stanford CVI iPS cell Biobank.

### Massively parallel reporter assays

Measured effects of single nucleotide substitution effects from saturation mutagenesis experiments across 29 regulatory elements were obtained from^40^ and across 131 elements from^9^. For each nucleotide, the mean substitution effect across all reported nucleotides was correlated (Pearson) with phyloP scores that were truncated such that negative values, which are indicative of possible acceleration, were set to zero. A Storey FDR^60^ was used to control for multiple comparisons. Regulatory effects from 27,017 common variants in the DHS elements investigated in this study were obtained from^9^. Variants with a reported FDR below 5% were defined as allele-specific. A generalized linear model with a binomial probability distribution was used to estimate the effects of constraint on allele-specific activity.

### Chromatin accessibility and histone modifications in non-humans

Chromatin accessibility from ATAC-seq in fibroblasts obtained from human and 4 non-human primates (chimpanzee, gorilla, orangutan and macaque) at 89,744 merged peaks with orthologous sequences in all 5 species were obtained from^42,75^. Counts were transformed to log_2_ counts per million (cpm), and FDR values from differential accessibility testing across any primate species were obtained^42^.

Histone modifications (H3K27ac) were also obtained from three matching cell types during corticogenesis for human, macaque, and mouse^43^. First, H3K27ac peaks at orthologous sequences from all species were obtained from the authors and filtered such that at least 200 bp of these peaks overlapped with a DHS element in this study. Next, DHS elements coordinates in GRCh38 were lifted over to each species and the maximum H3K27ac signal (cpm) at each element was calculated using the provided bigwig files. Spearman correlations between matching cell types were then computed for each pair of species stratified by the type of constraint on the DHS element.

### Reporting summary

Further information on research design is available in the Nature Portfolio Reporting Summary linked to this article.

## Online content

Any methods, additional references, Nature Portfolio reporting summaries, source data, extended data, supplementary information, acknowledgements, peer review information; details of author contributions and competing interests; and statements of data and code availability are available at 10.1038/s41586-023-06798-8.

### Supplementary information

Supplementary Information This file contains Supplementary Tables 1–3 and the legends for Supplementary Data 1–10 (see separate Excel file for the Supplementary Data).

Reporting Summary

Supplementary Data This file contains Supplementary Data 1–10.

## Data Availability

Primate assemblies have been deposited at the European Nucleotide Archive (ENA) under the accession PRJEB67744. The MSA and constraint tracks are available through the UCSC Genome Browser.
