# Supplementary material for: Identification of constrained sequence elements across 239 primate genomes
Source: Nature. 2023 Nov 29;625(7996):735–42. doi: 10.1038/s41586-023-06798-8 (PMC10808062; doi:10.1038/s41586-023-06798-8)
Supplement: Supplementary file 1 — This file contains Supplementary Tables 1–3 and the legends for Supplementary Data 1–10 (see separate Excel file for the Supplementary Data). [file 41586_2023_6798_MOESM1_ESM.docx]

**Supplemental Tables**

| Species | Mismatch-rate (bp^-1^) | Heterozygosity (bp^-1^) |
| --- | --- | --- |
| Callithrix jacchus | 0.0013 | 0.0012 |
| Cebus albifrons | 0.0033 | 0.0022 |
| Cercopithecus mitis | 0.0025 | 0.0017 |
| Colobus guereza | 0.0026 | 0.0013 |
| Daubentonia madagascariensis | 0.0007 | 0.0006 |
| Erythrocebus patas | 0.0023 | 0.0016 |
| Galago moholi | 0.0021 | 0.0019 |
| Carlito syrichta | 0.0016 | 0.0023 |
| Propithecus coquereli | 0.0052 | 0.0056 |
| Gorilla gorilla | 0.0017 | 0.0018 |
| Lemur catta | 0.003 | 0.0032 |
| Loris tardigradus | 0.0029 | 0.0029 |
| Macaca mulatta | 0.0031 | 0.0023 |
| Mandrillus sphinx | 0.0043 | 0.0045 |
| Microcebus murinus | 0.004 | 0.0042 |
| Nycticebus pygmaeus | 0.0009 | 0.0008 |
| Otolemur garnettii | 0.0026 | 0.0018 |
| Pan troglodytes | 0.0009 | 0.0009 |
| Papio anubis | 0.002 | 0.0018 |
| Pongo abelii | 0.0027 | 0.0028 |
| Pongo pygmaeus | 0.0019 | 0.0017 |
| Rhinopithecus roxellana | 0.0003 | 0.0004 |
| Saguinus midas | 0.0019 | 0.0024 |
| Sapajus apella | 0.0021 | 0.0016 |
| Theropithecus gelada | 0.0014 | 0.0005 |

**Supplementary Table S1**

Mismatch rates and heterozygosity estimates for all species assembled in this project for which a previously assembled reference genome was available for comparison.

| GO biological process complete | over/ under | fold enrichment | raw P-value | FDR |
| --- | --- | --- | --- | --- |
| antibacterial humoral response (GO:0019731) | + | 26.4 | 1.78E-09 | 2.14E-06 |
| antimicrobial humoral response (GO:0019730) | + | 13.75 | 5.57E-09 | 5.80E-06 |
| detection of chemical stimulus involved in sensory perception of smell (GO:0050911) | + | 11.17 | 5.48E-19 | 2.14E-15 |
| detection of chemical stimulus involved in sensory perception (GO:0050907) | + | 10.89 | 3.21E-20 | 5.00E-16 |
| sensory perception of smell (GO:0007608) | + | 10.53 | 2.09E-18 | 5.43E-15 |
| humoral immune response (GO:0006959) | + | 10.29 | 7.35E-10 | 1.04E-06 |
| detection of chemical stimulus (GO:0009593) | + | 10.2 | 1.57E-19 | 1.22E-15 |
| sensory perception of chemical stimulus (GO:0007606) | + | 9.74 | 4.88E-19 | 2.54E-15 |
| detection of stimulus involved in sensory perception (GO:0050906) | + | 9.46 | 9.81E-19 | 3.06E-15 |
| detection of stimulus (GO:0051606) | + | 8.08 | 1.03E-17 | 2.30E-14 |
| defense response to bacterium (GO:0042742) | + | 6.73 | 3.12E-06 | 1.87E-03 |
| sensory perception (GO:0007600) | + | 5.37 | 6.46E-13 | 1.12E-09 |
| G protein-coupled receptor signaling pathway (GO:0007186) | + | 5.31 | 1.15E-15 | 2.25E-12 |
| innate immune response (GO:0045087) | + | 4.21 | 1.40E-06 | 9.08E-04 |
| defense response to other organism (GO:0098542) | + | 3.77 | 6.51E-07 | 4.42E-04 |
| nervous system process (GO:0050877) | + | 3.66 | 3.09E-09 | 3.44E-06 |
| defense response (GO:0006952) | + | 2.79 | 2.74E-05 | 1.34E-02 |
| system process (GO:0003008) | + | 2.76 | 3.20E-07 | 2.50E-04 |
| response to other organism (GO:0051707) | + | 2.76 | 5.31E-05 | 2.30E-02 |
| response to external biotic stimulus (GO:0043207) | + | 2.75 | 5.46E-05 | 2.31E-02 |
| response to biotic stimulus (GO:0009607) | + | 2.67 | 8.38E-05 | 3.11E-02 |
| response to chemical (GO:0042221) | + | 1.93 | 2.54E-05 | 1.28E-02 |
| signal transduction (GO:0007165) | + | 1.78 | 5.76E-05 | 2.36E-02 |
| nitrogen compound metabolic process (GO:0006807) | - | 0.33 | 4.23E-07 | 3.14E-04 |
| primary metabolic process (GO:0044238) | - | 0.3 | 2.00E-08 | 1.95E-05 |
| multicellular organism development (GO:0007275) | - | 0.3 | 1.45E-04 | 4.92E-02 |
| metabolic process (GO:0008152) | - | 0.29 | 4.77E-10 | 7.44E-07 |
| organic substance metabolic process (GO:0071704) | - | 0.28 | 1.20E-09 | 1.56E-06 |
| organonitrogen compound metabolic process (GO:1901564) | - | 0.28 | 7.60E-06 | 4.23E-03 |
| cellular metabolic process (GO:0044237) | - | 0.27 | 4.54E-07 | 3.22E-04 |
| macromolecule metabolic process (GO:0043170) | - | 0.27 | 1.72E-07 | 1.42E-04 |
| transport (GO:0006810) | - | 0.26 | 1.00E-04 | 3.55E-02 |
| protein metabolic process (GO:0019538) | - | 0.26 | 6.73E-05 | 2.63E-02 |
| establishment of localization (GO:0051234) | - | 0.25 | 4.41E-05 | 1.97E-02 |
| localization (GO:0051179) | - | 0.22 | 2.18E-06 | 1.36E-03 |
| cellular component organization (GO:0016043) | - | 0.22 | 7.54E-08 | 6.54E-05 |
| cellular component organization or biogenesis (GO:0071840) | - | 0.21 | 2.74E-08 | 2.52E-05 |
| cellular localization (GO:0051641) | - | 0.15 | 1.09E-04 | 3.77E-02 |
| macromolecule modification (GO:0043412) | - | 0.14 | 7.15E-05 | 2.72E-02 |
| nervous system development (GO:0007399) | - | < 0.01 | 1.70E-05 | 9.16E-03 |
| positive regulation of biosynthetic process (GO:0009891) | - | < 0.01 | 2.52E-05 | 1.31E-02 |
| positive regulation of RNA metabolic process (GO:0051254) | - | < 0.01 | 9.46E-05 | 3.43E-02 |
| positive regulation of cellular biosynthetic process (GO:0031328) | - | < 0.01 | 3.91E-05 | 1.85E-02 |
| positive regulation of macromolecule biosynthetic process (GO:0010557) | - | < 0.01 | 6.09E-05 | 2.44E-02 |
| gene expression (GO:0010467) | - | < 0.01 | 6.62E-06 | 3.82E-03 |
| positive regulation of nucleobase-containing compound metabolic process (GO:0045935) | - | < 0.01 | 4.02E-05 | 1.84E-02 |

**Supplementary Table S2**

GO-term enrichment of GO biological processes for genes with primate-specific constraint. Two-sided Fisher’s Exact Test, Benjamini-Hochberg false-discovery rate. Results are significant at a false discovery rate of 5%.

| GO biological process complete | over/ under | fold enrichment | raw P-value | FDR |
| --- | --- | --- | --- | --- |
| homophilic cell adhesion via plasma membrane adhesion molecules (GO:0007156) | + | 7.92 | 2.26E-09 | 3.52E-05 |
| cell-cell adhesion via plasma-membrane adhesion molecules (GO:0098742) | + | 5.4 | 1.05E-07 | 3.27E-04 |
| anterior/posterior pattern specification (GO:0009952) | + | 5.1 | 7.44E-06 | 1.66E-02 |
| regionalization (GO:0003002) | + | 3.51 | 2.12E-05 | 3.30E-02 |
| cell-cell adhesion (GO:0098609) | + | 3.13 | 1.72E-05 | 2.98E-02 |
| nervous system development (GO:0007399) | + | 2.24 | 8.81E-09 | 4.59E-05 |
| system development (GO:0048731) | + | 1.92 | 4.98E-09 | 3.89E-05 |
| multicellular organism development (GO:0007275) | + | 1.83 | 1.31E-08 | 5.10E-05 |
| anatomical structure development (GO:0048856) | + | 1.56 | 3.11E-06 | 8.09E-03 |
| developmental process (GO:0032502) | + | 1.49 | 1.23E-05 | 2.40E-02 |
| multicellular organismal process (GO:0032501) | + | 1.41 | 2.19E-05 | 3.10E-02 |

**Supplementary Table S3**

GO-term enrichment of GO biological processes for genes containing CDS exons that overlap primate-UCEs. Two-sided Fisher’s Exact Test, Benjamini-Hochberg false-discovery rate. Results are significant at a false discovery rate of 5%.

**Supplemental Data (external files)**

**Supplemental Data S1**: Summary and source of assemblies for all species included in this study.

**Supplemental Data S2**: Protein-coding exons and genes with primate-specific constraint.

**Supplemental Data S3**: DHS with selective constraint across all mammals or specific to primates.

**Supplemental Data S4**: TF footprints with selective constraint across all mammals or specific to primates.

**Supplemental Data S5**: Coordinates of ultraconserved elements in primates.

**Supplemental Data S6**: Relative activity and fold-change data for luciferase assays

**Supplemental Data S7**: Correlations between phyloP constraint metrics and saturation mutagenesis massively parallel reporter assays.

**Supplemental Data S8**: Fine-mapped variants in primate-specific constrained protein-coding exons.

**Supplemental Data S9**: Fine-mapped variants in primate-specific constrained DHS.

**Supplemental Data S10**: Fine-mapped variants in primate-specific constrained TF footprints.
