# Supplementary material for: Identification of constrained sequence elements across 239 primate genomes
Source: Nature. 2023 Nov 29;625(7996):735–42. doi: 10.1038/s41586-023-06798-8 (PMC10808062; doi:10.1038/s41586-023-06798-8)
Supplement: Supplementary file 2 — Reporting Summary [file 41586_2023_6798_MOESM2_ESM.pdf]

nature portfolio

Corresponding author(s): Kyle Farh
Jeffrey Rogers
Tomas Marques Bonet

Last updated by author(s): Oct 9, 2023

# Reporting Summary

Nature Portfolio wishes to improve the reproducibility of the work that we publish. This form provides structure for consistency and transparency in reporting. For further information on Nature Portfolio policies, see our Editorial Policies and the Editorial Policy Checklist.

## Statistics

For all statistical analyses, confirm that the following items are present in the figure legend, table legend, main text, or Methods section.

| n/a | Confirmed | |
|---|---|---|
| ☐ | ☒ | The exact sample size (*n*) for each experimental group/condition, given as a discrete number and unit of measurement |
| ☒ | ☐ | A statement on whether measurements were taken from distinct samples or whether the same sample was measured repeatedly |
| ☐ | ☒ | The statistical test(s) used AND whether they are one- or two-sided<br>*Only common tests should be described solely by name; describe more complex techniques in the Methods section.* |
| ☐ | ☒ | A description of all covariates tested |
| ☐ | ☒ | A description of any assumptions or corrections, such as tests of normality and adjustment for multiple comparisons |
| ☐ | ☒ | A full description of the statistical parameters including central tendency (e.g. means) or other basic estimates (e.g. regression coefficient) AND variation (e.g. standard deviation) or associated estimates of uncertainty (e.g. confidence intervals) |
| ☐ | ☒ | For null hypothesis testing, the test statistic (e.g. *F*, *t*, *r*) with confidence intervals, effect sizes, degrees of freedom and *P* value noted<br>*Give P values as exact values whenever suitable.* |
| ☒ | ☐ | For Bayesian analysis, information on the choice of priors and Markov chain Monte Carlo settings |
| ☐ | ☒ | For hierarchical and complex designs, identification of the appropriate level for tests and full reporting of outcomes |
| ☐ | ☒ | Estimates of effect sizes (e.g. Cohen's *d*, Pearson's *r*), indicating how they were calculated |

*Our web collection on statistics for biologists contains articles on many of the points above.*

## Software and code

Policy information about availability of computer code

| | |
|---|---|
| Data collection | No software was used for data collection. |
| Data analysis | Megahit (v1.2.9)<br>RepeatMasker (v4.1.2-p1)<br>Cactus (v2.1.1)<br>minimap2 (v2.17-r941)<br>PHAST (v1.5)<br>FINEMAP (v1.4)<br>Su5iE (v0.12.10)<br>LDstore (v2)<br>motifbreakR (v3.16)<br>Panther (v16)<br>IQTree (v1.6.12)<br>VEP (v85)<br>GenomicRanges (v3.1.7)<br>Haltoos (v2.2) |

For manuscripts utilizing custom algorithms or software that are central to the research but not yet described in published literature, software must be made available to editors and reviewers. We strongly encourage code deposition in a community repository (e.g. GitHub). See the Nature Portfolio guidelines for submitting code & software for further information.

## Data

Policy information about availability of data

All manuscripts must include a data availability statement. This statement should provide the following information, where applicable:

- Accession codes, unique identifiers, or web links for publicly available datasets
- A description of any restrictions on data availability
- For clinical datasets or third party data, please ensure that the statement adheres to our policy

Primate assemblies have been deposited at ENA under the accession PRJEB67744. The MSA and constraint tracks are available through the UCSC genome browser.

## Research involving human participants, their data, or biological material

Policy information about studies with human participants or human data. See also policy information about sex, gender (identity/presentation), and sexual orientation and race, ethnicity and racism.

| | |
|---|---|
| Reporting on sex and gender | not applicable |
| Reporting on race, ethnicity, or other socially relevant groupings | not applicable |
| Population characteristics | not applicable |
| Recruitment | not applicable |
| Ethics oversight | not applicable |

Note that full information on the approval of the study protocol must also be provided in the manuscript.

# Field-specific reporting

Please select the one below that is the best fit for your research. If you are not sure, read the appropriate sections before making your selection.

☒ Life sciences ☐ Behavioural & social sciences ☐ Ecological, evolutionary & environmental sciences

For a reference copy of the document with all sections, see nature.com/documents/nr-reporting-summary-flat.pdf

# Life sciences study design

All studies must disclose on these points even when the disclosure is negative.

| | |
|---|---|
| Sample size | Sample size was based on available primate genomes. |
| Data exclusions | No data was excluded from analyses. |
| Replication | All wet-lab experiments were replicated 3 or more times. All attempts at replication were successful. |
| Randomization | Randomization was not necessary for this study, as genomic sequences were derived from a single individual. |
| Blinding | Investigators were not blinded to the species names or genome sequences. Blinding was was not necessary for this study, as genomic sequences were derived from a single individual. |

# Reporting for specific materials, systems and methods

We require information from authors about some types of materials, experimental systems and methods used in many studies. Here, indicate whether each material, system or method listed is relevant to your study. If you are not sure if a list item applies to your research, read the appropriate section before selecting a response.

## Materials & experimental systems

| n/a | Involved in the study |
|---|---|
| ☒ | ☐ Antibodies |
| ☐ | ☒ Eukaryotic cell lines |
| ☒ | ☐ Palaeontology and archaeology |
| ☒ | ☐ Animals and other organisms |
| ☒ | ☐ Clinical data |
| ☒ | ☐ Dual use research of concern |
| ☒ | ☐ Plants |

## Methods

| n/a | Involved in the study |
|---|---|
| ☒ | ☐ ChIP-seq |
| ☒ | ☐ Flow cytometry |
| ☒ | ☐ MRI-based neuroimaging |

# Eukaryotic cell lines

Policy information about cell lines and Sex and Gender in Research

| | |
|---|---|
| Cell line source(s) | Human iPSCs were obtained from the Stanford CVI iPSC Biobank. |
| Authentication | Immunofluresence assay was performed to check the expression of stem cell markers such as NANOG, POUSF1 and SOX2. SNP karyotyping was tested through HuCytoSNP-12 chip (Illumina), and CNV and SNP visualization was performed using KaryoStudio v1.4 (Illumina). |
| Mycoplasma contamination | We confirmed that the cell line was negative for mycoplasma contamination using MycoAlertTM PLUS Mycoplasma Detection Kit (Lanza) |
| Commonly misidentified lines (See ICLAC register) | No commonly misidentified cell lines were used. |

# Plants

| | |
|---|---|
| Seed stocks | No plant materials were used in this study. |
| Novel plant genotypes | No plant materials were used in this study. |
| Authentication | No plant materials were used in this study. |
